# Supplementary material for: Cancer mutationscape: revealing the link between modular restructuring and intervention efficacy among mutations
Source: NPJ Syst Biol Appl. 2024 Jul 13;10:74. doi: 10.1038/s41540-024-00398-6 (PMC11246485; doi:10.1038/s41540-024-00398-6)
Supplement: Supplementary file 1 — Supplementary File [file 41540_2024_398_MOESM1_ESM.pdf]

Supplementary material

Aggression scores

Uncontrolled Trajectory Approximations																
	N.I.	KRAS	TP53	CycD	SMAD	T-K	C-K	S-K	T-C	T-S	C-S	T-C-K	T-S-K	C-S-K	T-C-S	T-C-S-K
Aut_c	0.509	0.531	0.964	0.818	0.528	0.974	0.827	0.356	0.953	0.955	0.817	0.97	0.967	0.82	0.962	0.961
Apo_c	0.359	0.356	0.021	0.109	0.343	0.011	0.118	0.479	0.025	0.035	0.105	0.018	0.013	0.1	0.028	0.017
Pro_c	0.213	0.209	0.56	0.017	0.211	0.555	0.013	0.24	0.018	0.92	0.012	0.015	0.944	0.018	0.01	0.014

Supplementary Table 1: *Expression Approximations*. This table records the approximate phenotype expressions for the PCC in Figure 1. Given 1,000 random initial states, these results show trajectory approximations after 300 time steps (i.e. function updates) with 1% noise.

We derived aggressiveness scores for each mutation combination using long-term trajectory approximations. Simulations were run using 1000 random initializations, 300 time steps, and 1% noise to achieve an approximate probability of phenotype expression. In Table, 1, we see that the non-induced (N.I.) system showed levels of 51% autophagy, 36% apoptosis, and 21% proliferation. The heat maps in the manuscript are sorted with column-wise mutation groups and used to compare cancer cell autophagy and proliferation while giving a negative weight ($\omega = -1$) to apoptosis. The row label "Same" indicates that the same weight was given to both autophagy and proliferation (used value $\omega = 2$ for both), "High/Low" indicates a high weight for autophagy ($\omega = 10$) but a low weight for proliferation ($\omega = 2$), and "Low/High" indicates a low weight for autophagy ($\omega = 2$) but a high weight for proliferation ($\omega = 10$). Thus, scores were calculated using:

$$\text{Score} = \text{Aut}_c \times \omega_1 + \text{Pro}_c \times \omega_2 + \text{Apop}_c \times (-1) \quad \text{where} \quad \omega_{1,2} \in \{2, 10\}$$

Scaling of the heat map ranges orange (low score) to red (high score) based on the maximum and minimum values in each table. However, blue shading (i.e. cold) indicates a negative score, which is interpreted as successful depletion of aggression. See [1] sections 2.3 and 4.4 for more details.

Lastly, we justify the positive weight given to autophagy, which is a natural process where cells heal themselves. The cell will break down any damaged or unnecessary components, and it will reallocate the nutrients from these processes to those that are essential. However, studies have shown that autophagy is required for pancreatic tumor growth [2]. Autophagy can help tumors overcome conditions such as hypoxia and nutrient deprivation. Within tumors, cells can exist under hypoxic conditions. If activated autophagy is then suppressed by deletion of Beclin 1, studies have shown increased cell death. It has also been observed that autophagy is increased in KRAS mutated cells, and aids in survival of the cancer cells while experiencing nutrient starvation. Further, animal studies have shown that autophagy contributes to tumor-cell survival by enhancing stress tolerance and supplying nutrients to meet the metabolic demands of tumors. Once suppression of autophagy occurred, there was an observance of tumor-cell death [3].

Note: our aggression scores are based on combinations autophagy, apoptosis, and proliferation, merely one method among many for estimating aggression. Moreover, the attractor analysis (see [4, 1] indicated that certain mutation combinations yield a large basin for attractors with both autophagy and proliferation expression. It is likely that modular structure alone is not enough to determine aggression and target cardinality. Rather, it should be used alongside other analyses.

Boolean pancreatic cancer model and functions

Cytokines	(Cyan)
VEGF	NFκBs | STATp | NFκBp
EGF	cJUNp
bFGF	ERKs | ERKp
TNFα	TNFα
PDGFBB	SMADs | SMADp
Thiazolidinedione	Thiazolidinedione
TGFβ1	SMADs | SMADp
IFNγ	IFNγ
Pancreatic Stellate Cell	(Green)
VEGFRs	VEGF
EGFRs	EGF
FGFRs	bFGF
TNFRs	TNFα
PDGFBBRs	PDGFBB
PPARγs	Thiazolidinedione
TGFRs	TGFβ1
IFNGRs	IFNγ
RASs	((VEGFRs) | (EGFRs) | (FGFRs))
PIK3s	((PDGFBBRs) | (RASs))
SMADs	TGFRs
STATs	IFNGRs
RAFs	RASs
NFκBs	((TNFRs) | (AKTs))
P38s	((MEKs) | (P53s))
MEKs	RAFs
PIP3s	($\sim$ PTENs) & (PIK3s)
P53s	($\sim$ MDM2s) & (P38s)
PTENs	P53s
AKTs	PIP3s
ERKs	(PDGFBBRs) | (MEKs)
AP1s	ERKs
P21s	P53s
MDM2s	((AKTs) | (P53s))
Apos	P53s
Pros	($\sim$ P21s) & (AP1s)
Migs	(AKTs) & (AP1s) & (Acts)
Acts	SMADs & (($\sim$STATs) | ($\sim$PPARγs)) & (NFκBs | AP1s)
Pancreatic Cancer Cell	(Purple)
EGFRc	EGF | HER2c
FGFRc	bFGF
TGFRc	TGFβ1
HER2c	HER2c
JAK1c	HER2c

PIK3c	(EGFRc) | (RASc)
RASc	(EGFRc) | (FGFRc)
SMADc	TGFRc
STATc	JAK1c
PIP3c	($\sim$ PTENc) & (PIK3c)
RAFc	RASc
P21c	($\sim$ STATc) & ((SMADc) | (P53c))
MEKc	RAFc
NFκBc	AKTc
AKTc	PIP3c
PTENc	P53c
ERKc	MEKc
E2Fc	($\sim$ RBc)
cJUNc	(ERKc) | (JNKc)
CyclinDc	($\sim$ P21c) & (NFκBc)
RBc	($\sim$ CyclinDc)
BCLXLc	($\sim$ P53c) & ((NFκBc) | (STATc) | (AKTc) | (JNKc))
JNKc	MEKc
mTORc	($\sim$ cJUNc) & (AKTc)
BAXc	($\sim$ BCLXLc)
Beclin1c	($\sim$ BCLXLc) & ($\sim$ CASPc)
MDM2c	($\sim$ E2Fc) & (AKTc | P53c)
P53c	($\sim$ MDM2c)
CyclinEc	($\sim$ P21c) & (E2Fc)
CASPc	($\sim$ NFκBc) & ((P53c) | (Beclin1c) | (BAXc))
Autc	($\sim$ mTORc) & ((NFκBc) | (Beclin1c))
Apoc	CASPc
Proc	(CyclinEc) & ((JNKc) | (cJUNc))

Supplementary Table 2: *Boolean functions for the whole pancreatic cancer model.* Each function indicates the next state of the node in terms of the current states of said nodes' regulators. Activation is written as OR statements, while suppression is written as AND NOT. The exception to this rule is PCC proliferation, because of its upstream signaling.

Cytokines	(Cyan)
TNFα	TNFα
Thiazolidinedione	Thiazolidinedione
TGFβ1	TGFβ1
IFNγ	IFNγ
Stellate Cell	(Green)
RASs	((NFκBs) | (STATp) | (PIP3p) | (RASp) | (ERKs))
NFκBs	((TNFα) | (PIP3s))
PIP3s	($\sim$ P53s) & ((TGFβ1) | (RASs))
P53s	($\sim$ PIP3s) & ($\sim$ P53s) & (RASs)
ERKs	(TGFβ1) | (RASs)
Pros	($\sim$ P53s) & (ERKs)
Migs	(PIP3s) & (ERKs) & (Acts)
Acts	(TGFβ1) & (($\sim$ IFNγ) | ($\sim$ Thiaz.)) & ((NFκBs) | (ERKs))
Pancreatic Cell	(Purple)
HER2p	HER2p
RASp	(RASp) | (HER2p) | (ERKs)
STATp	HER2p
PIP3p	($\sim$ P53p) & ((RASp) | (HER2p))
P21p	($\sim$ STATp) & ((TGFβ1) | (P53p))
BCLXLp	($\sim$ P53p) & ((PIP3p) | (STATp) | (RASp))
P53p	$\sim$ (((P21p) | ($\sim$ PIP3p)) & ((PIP3p) | (P53p)))
CASPp	($\sim$ PIP3p) & ((P53p) | ($\sim$ BCLXLp))
Autp	((RASp) | ($\sim$ PIP3p)) & ((PIP3p) | (($\sim$ BCLXLp) & ($\sim$ CASPp)))
Prop	($\sim$ P21p) & (PIP3p) & (RASp)

Supplementary Table 3: *Boolean functions for the reduced pancreatic cancer model.* Each function indicates the next state of the node in terms of the current states of said nodes' regulators. Activation is written as OR statements, while suppression is written as AND NOT. Functions maintain the rules from the whole model by substituting values from the deleted nodes.

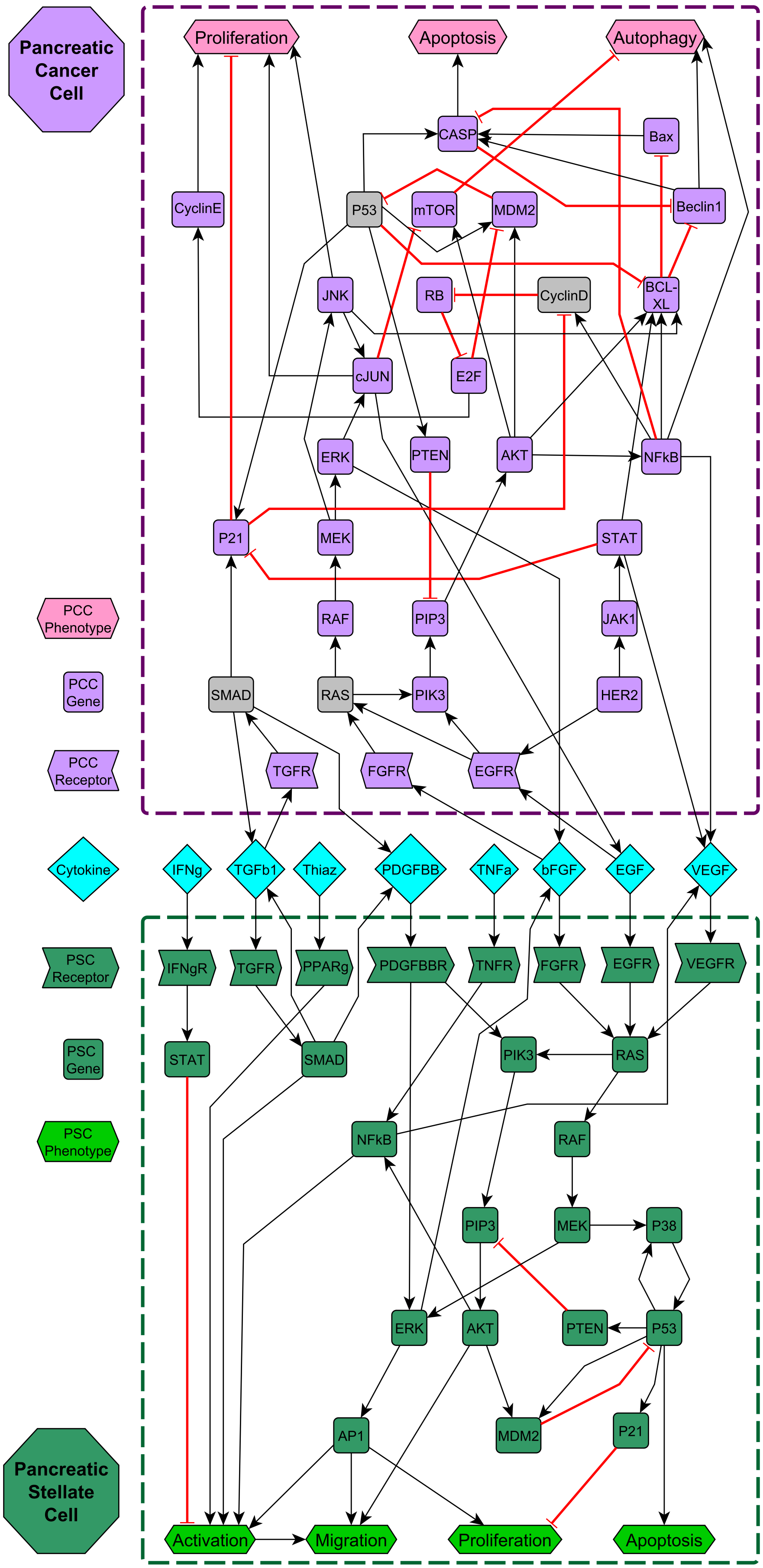

Supplementary Figure 1: *Gene regulatory network model of pancreatic cancer.* Shapes and colors of nodes indicate their function and cell type (respectively), as shown in the legend. Black barbed arrows indicate signal expression, while red bar arrows indicate suppression. Grey nodes located in the PCC indicate prevalent mutant genes [4, 5].

Tables and Graphs

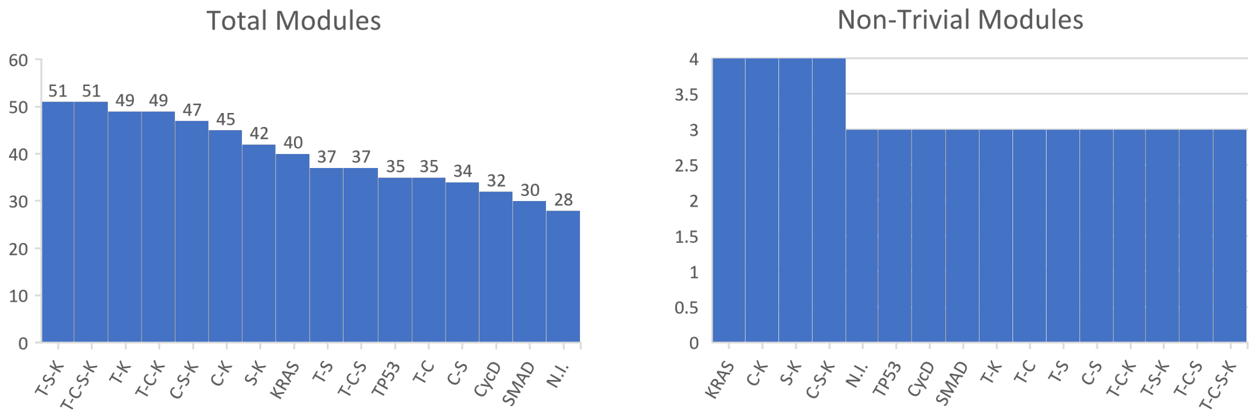

(a) Total module counts

(b) Non-trivial module counts

Supplementary Figure 2: *Module counts*

Supplementary Figure 3: *PC condensation graphs.* Included are all condensation graphs for each mutation combination. These are directed, acyclic graphs that are topologically sorted, and whose nodes represent the strongly connected components of Figure 1. Colors of nodes are based on the components they represent, and node numbers correspond to bin numbers (see data files in the repository).

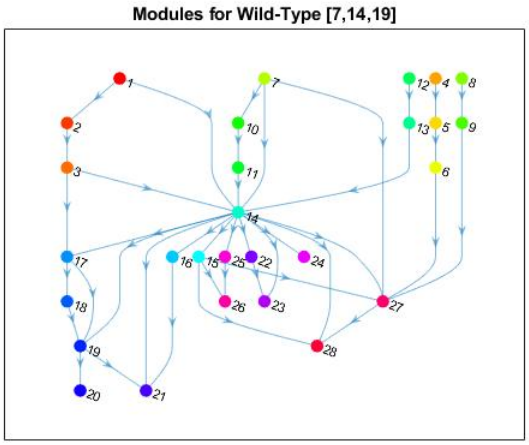

(a) Wild-type condensation graph.

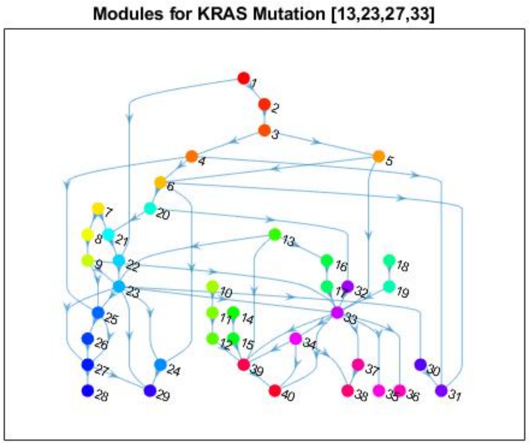

(b) KRAS condensation graph.

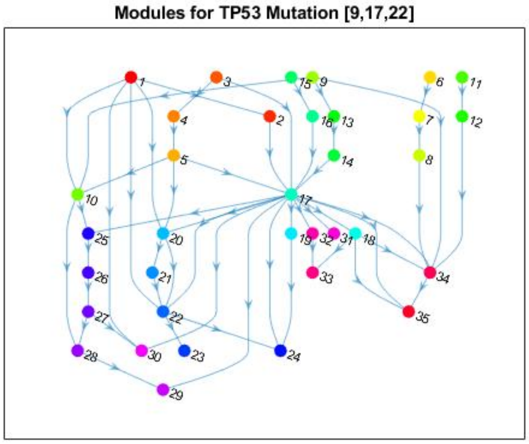

(c) TP53 condensation graph.

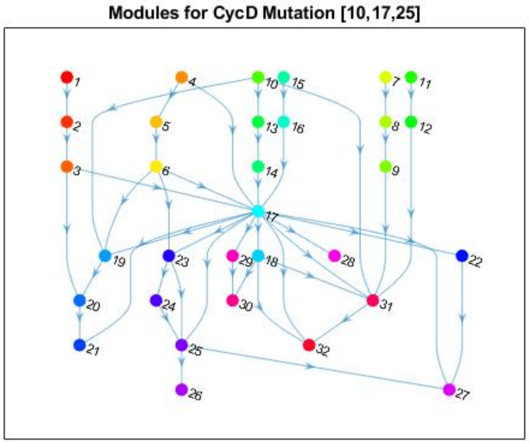

(d) CyclinD condensation graph.

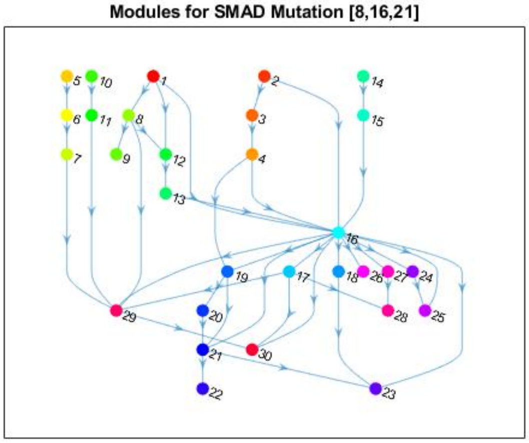

(e) SMAD condensation graph.

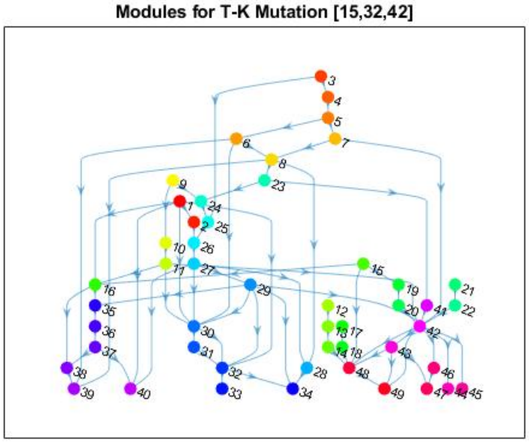

(f) T/K condensation graph.

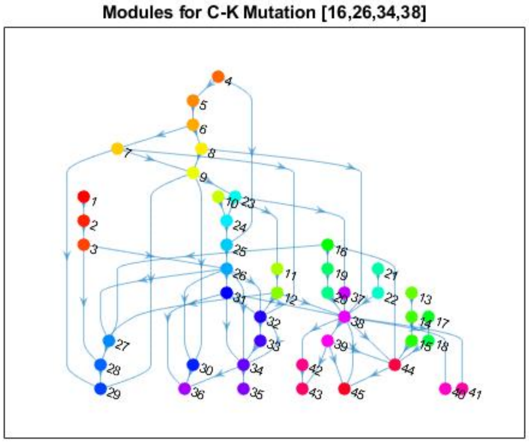

(g) C/K condensation graph.

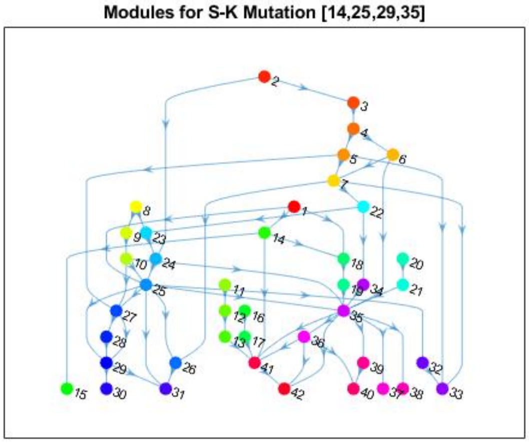

(h) S/K condensation graph.

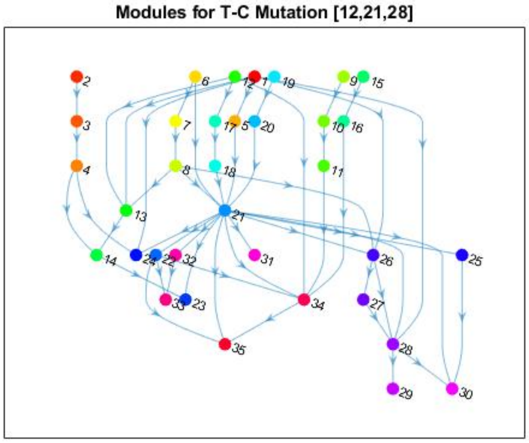

(i) T/C condensation graph.

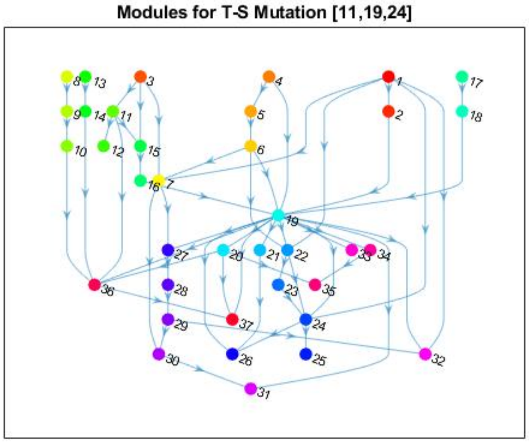

(j) T/S condensation graph.

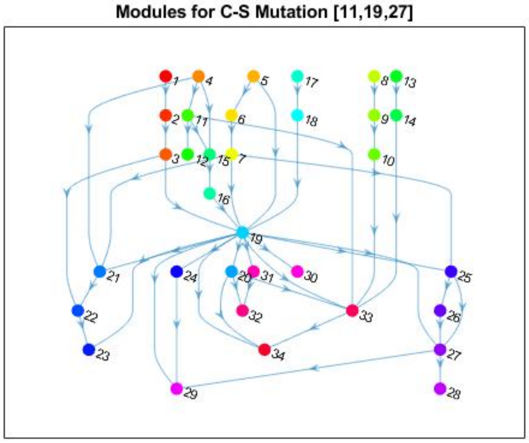

(k) C/S condensation graph.

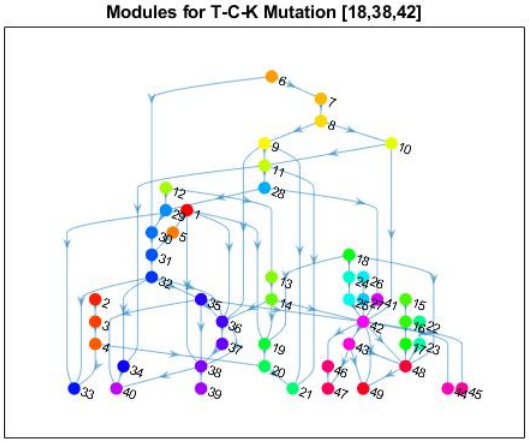

(l) T/C/K condensation graph.

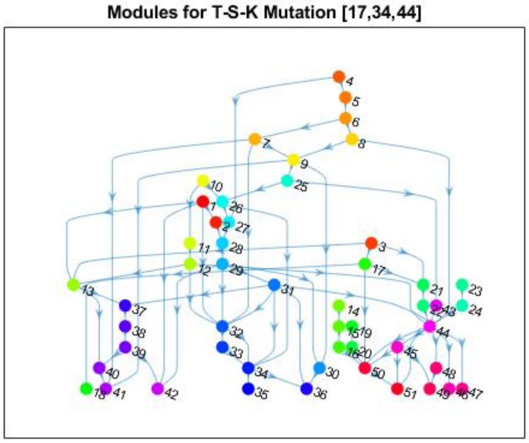

(m) T/S/K condensation graph.

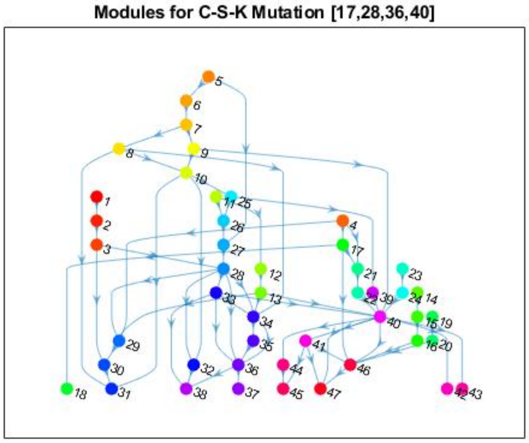

(n) C/S/K condensation graph.

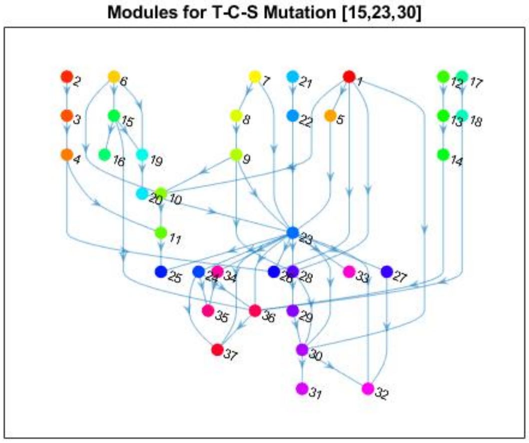

(o) T/C/S condensation graph.

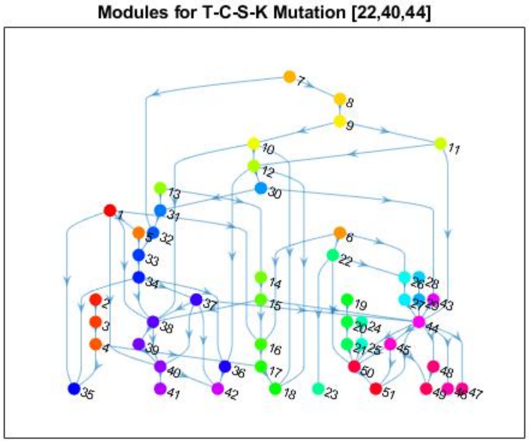

(p) T/C/S/K condensation graph.

References

[1] Plaugher, D., Aguilar, B. & Murrugarra, D. Uncovering potential interventions for pancreatic cancer patients via mathematical modeling. *Journal of Theoretical Biology* **548**, 111197 (2022). URL https://www.sciencedirect.com/science/article/pii/S0022519322001953.

[2] Yang, S. *et al.* Pancreatic cancers require autophagy for tumor growth. *Genes & Development* **25**, 717–729 (2011). URL http://genesdev.cshlp.org/content/25/7/717.abstract. http://genesdev.cshlp.org/content/25/7/717.full.pdf+html.

[3] Yun, C. W. & Lee, S. H. The roles of autophagy in cancer. *International Journal of Molecular Sciences* **19** (2018). URL https://www.mdpi.com/1422-0067/19/11/3466.

[4] Plaugher, D. & Murrugarra, D. Modeling the pancreatic cancer microenvironment in search of control targets. *Bulletin of Mathematical Biology* **83** (2021).

[5] Wang, Q. *et al.* Formal modeling and analysis of pancreatic cancer microenvironment. In Bartocci, E., Lio, P. & Paoletti, N. (eds.) *Computational Methods in Systems Biology*, 289–305 (Springer International Publishing, Cham, 2016).
